# Assessing the Correlation between Blood Trace Element Concentrations, Picky Eating Habits, and Intelligence Quotient in School-Aged Children

**DOI:** 10.3390/children10071249

**Published:** 2023-07-20

**Authors:** Abdullah A. Saati, Heba M. Adly

**Affiliations:** Community Medicine and Pilgrims Healthcare Department, Faculty of Medicine, Umm Al-Qura University, Makkah 21955, Saudi Arabia; aaasaati@uqu.edu.sa

**Keywords:** children, trace elements, zinc deficiency, iron deficiency, children IQ, eating habits

## Abstract

Introduction: Inadequate levels of iron, zinc, and copper have been linked to growth impairment and cognitive and motor development deficits. The objective of this study is to examine the deficiencies of trace elements and their correlation with selective eating patterns and the intelligence quotient (IQ) of children. Methods and Patients: The cross-sectional analysis involved 430 children aged between 7 and 10 years. Blood samples were analyzed using Inductively Coupled Plasma Mass Spectrometry (ICP-MS) to measure the trace elements levels. Children’s IQs were assessed using Raven’s Standard Progressive Matrices. Results: Among the sample group, 20.3% exhibited iron deficiency, 42.5% had zinc deficiency, and 14% had insufficient copper levels. Single trace element deficiency was observed in 56.9% of the children, while 66.7% showed coexisting deficiencies of iron and zinc. Children with lower development levels exhibited significantly lower serum zinc levels compared to those with higher development levels (76.78 ± 10.67 vs. 81.14 ± 10.19 μg/dL). The analysis reveals that picky eaters had lower serum iron levels (76.59 ± 10.42 μg/dL) and higher serum copper levels (123.74 ± 13.45 μg/dL). Conclusion: A strong association was observed between zinc deficiency, picky eating habits, and lower developmental stages. The findings underscore the importance of monitoring nutritional status in children, given the significant implications for their cognitive development.

## 1. Introduction

Micronutrient deficiencies, including trace elements, are a significant global nutritional issue affecting both developing and developed countries [1,2,3]. Trace elements play essential roles in various bodily functions, such as immune response and antioxidant activity, and serve as crucial components or enzyme cofactors in metabolism [4,5]. Deficiencies in trace elements are common in several developing countries due to increased physiological demands and limited intake of nutrient-rich foods [6].

Recent research has highlighted not only iron deficiency but also deficiencies in zinc and copper, which have been linked to various health problems, including growth impairment and disorder [7,8,9,10]. Iron deficiency is associated with cognitive and motor development delays, and iron deficiency anemia has been shown to negatively impact cognitive functioning and psychomotor development [11]. Zinc deficiency can affect cognitive development by influencing neuropsychological behavior and motor development [12], While an inadequate intake of copper can result in hematological and neurological disorders [13].

Preschool children have heightened nutritional needs due to their rapid growth, and malnutrition during this phase can have lasting developmental repercussions, including delays in development, psychomotor deceleration, and behavioral disorders [14,15]. Insufficient dietary patterns, including selective eating or insufficient feeding practices by caregivers, can result in the overconsumption of certain foods and imbalanced diets, leading to overweightness and inadequate intake of trace elements [16,17,18,19,20]. Picky eating behaviors are characterized by the refusal to eat certain foods, neophobia, and other irregular eating habits [21]. Children with picky eating habits often display stunted growth, are underweight, have a lower body mass index (BMI) [22,23], and are more prone to experiencing diminished attention spans, impaired interpersonal relationships, and reduced levels of physical activity [24,25].

A study discovered a notable link between poverty and iron deficiency anemia among children of elementary school age [26]. A recent longitudinal study reported a high prevalence of iron deficiency at age 1, which decreased by age 3, with zinc deficiency being rare before age 3 [27]. The correlation between deficiencies in trace elements, picky eating behaviors, developmental stage, and physical activity levels remains unclear, especially among children in the developmental phase [28].

Lead and mercury are well-known trace elements with neurotoxic effects [29]. Exposure to lead via contaminated water or old paint can lead to developmental delays and behavioral issues in children. Similarly, industrial processes release mercury, which can be stored in the body and trigger significant health problems, including cognitive impairment [30].

Studies have shown that the timing and duration of exposure to these toxic elements during crucial stages of brain development can have long-lasting negative effects on cognitive function, including lower IQ scores [31]. Despite growing evidence of the potential harm, exposure to trace elements remains a prevalent concern, especially in urban areas and regions with heavy industrial activities. It is imperative to mitigate children’s exposure to these elements to safeguard their cognitive development [32].

The objective of this study is to evaluate the prevalence of zinc, iron, and copper deficiencies and investigate their correlation with selective eating behaviors and IQ levels among school-aged children (7–10 years old) in the Makkah region of Saudi Arabia.

## 2. Materials and Methods

### 2.1. Study Design and Sample Selection

To examine the serum levels of trace elements (zinc, iron, copper, mercury) and their correlations with predictors of outcomes (such as selective eating behaviors, development, and physical activity), a descriptive cross-sectional study was carried out involving children aged 7–10 years.

A sample size of 500 children was selected randomly for participation in this study, following the parents’ provision of informed consent. To maintain a consistent and reliable range of cognitive abilities within the same age group, children below 7 years or above 10 years were excluded, resulting in a final sample of 430 children. Trained physicians administered a structured questionnaire to gather demographic and socioeconomic information, including age, parents’ occupation, smoking, and drug use during pregnancy. The educational levels of the mothers were categorized as primary or below high school and university level or above.

Children who had chronic illnesses that could potentially impact eating habits, physical activity, or nutritional status were excluded from this study. Face-to-face interviews were conducted with parents or caregivers to collect sociodemographic data and evaluate picky eating behaviors, dietary habits, development, and physical activity using structured questionnaires. The questionnaire consisted of closed-ended categorical questions pertaining to children’s food preferences, parent–child interactions during mealtimes, language proficiency, developmental behaviors, and medical records within the past year. Detailed instructions were provided to parents on how to complete the questionnaires.

The main objective of this study was to assess the prevalence of deficiencies in trace elements (iron, zinc, copper, mercury) and investigate the associations between serum levels of trace elements and picky eating behaviors, developmental status, and physical activity levels. A secondary objective was to compare the connections between trace element deficiencies and picky eating behaviors, lower developmental status, or reduced physical activity levels while also measuring the strength of correlation for these categorical variables. The questionnaire employed in this study was adapted from “The framework for the United Kingdom Department of Health Survey of the Diets of British School Children” [33].

The study protocol received approval from the Ethics Review Board for Human Studies at the Faculty of Medicine, Umm Al-Qura University, with reference number YMRD310822, on 23 September 2022. The approval was granted in compliance with the guidelines of the Saudi National Committee for Bioethics (HABO-02-K-012).

### 2.2. Evaluation of the Children’s Selective Eating Habits

The assessment of selective eating behaviors in children was conducted using a structured closed-ended questionnaire. The questionnaire consisted of two parts: one focusing on food preferences and the other addressing parental feeding behaviors and eating behaviors of the children. The questionnaire was adapted from “The framework for the United Kingdom Department of Health Survey of the Diets of British School Children” [33]. The section assessing food preferences included an analysis of the child’s meals and their favorites towards seven diet groupings: grains, protein-rich foods, vegetables, fruits, dairy products, fats and oils, and snacks and sweets. Participants rated their preferences on a 5-point scale, varying from “like very much” to “dislike very much”.

The assessment of selective eating behaviors in this study involved four specific questions: limited food variety, reluctance to consume regular meals, hesitancy to try new foods, and exclusion of food groups from the seven major categories. Participants provided responses on a scale ranging from never (1) to always (5). Mean scores were computed for each subscale, where higher scores indicated a greater frequency of the respective behavior. Selective eating was defined in this study as a positive response of “always” to at least one item on the selective eating behaviors questionnaire, aligning with previous research [34,35,36,37,38,39].

### 2.3. Blood Trace Elements Analysis

#### 2.3.1. Samples Collection

During the morning, a blood sample of 10 mL was collected from each of the participating healthy children. The collected blood was divided into two parts: one part was collected in containers with EDTA to prevent coagulation, while the other part was placed in uncoated centrifuge tubes. The samples in the uncoated tubes were allowed to coagulate and then centrifuged at a speed of 3000 revolutions per minute for 15 min, approximately 1006× *g*. Following centrifugation, the serum was carefully separated. The samples were stored in a deep freezer at a temperature of −20 °C until the day of analysis.

#### 2.3.2. Laboratory Analysis Methods

The trace elements, namely iron, zinc, copper, and mercury, were analyzed using an Inductively Coupled Plasma Mass Spectrometry (ICP-MS) instrument (Perkin Elmer 7300, Perkin Elmer, Waltham, MA, USA), following the manufacturer’s instructions as shown in Table 1. The instrument was operated under the specified parameters: carrier gas (argon, 99.999% purity) at a flow rate of 0.8 L/min, plasma gas (argon, 99.999% purity) at 13 L/min, auxiliary gas (argon, 99.999% purity) at 0.8 L/min, pump rate at 1.5 mL/min, and power at 1055 KW. The recovery rates for the metal elements consistently exceeded 95%, and the detection limits for mercury were equal to or less than 3 ng/m^3^.

Stringent adherence to Quality Assurance and Quality Control (QA/QC) procedures were implemented to guarantee trustworthy outcomes. Regular analysis of a control sample was performed to assess the accuracy and precision of the analysis. Each sample was analyzed in triplicate to ensure reproducibility and linearity of the results. A linear calibration curve was constructed using a blank and a five-point calibration curve, encompassing concentrations spanning from 0.01 to 1.0 ppm for all trace elements.

To ensure instrument accuracy, external calibration was performed using ICP Element Standard Solutions VI CertiPUR 10 mg/L from Perkin Elmer, USA. The calibration solutions were meticulously prepared and stored in clean polypropylene vessels that were pre-cleaned with a 10% HNO_3_ solution and rinsed with ultra-pure water produced by a Millipore Mill-Q System, which had a resistivity of 18.2 Ώ cm. Calibration solutions were prepared on the same day as the analysis and shared the same acid matrix as the sample and blank solutions. This approach was implemented to minimize variability and bias in the analytical determination.

#### 2.3.3. Measurement of Children’s IQ Score

The intellectual abilities of the participating children were assessed using Raven’s Standard Progressive Matrices (SPM) [40]. The Raven’s SPM is a psychometric instrument that consists of five sets, comprising a total of 60 tasks. These tasks are designed to evaluate various cognitive abilities, including discrimination, analogy comparison, reasoning based on relationships, relational series, and abstract thinking. The complexity of the tasks progressively increases within each set, with a maximum potential score of 60. Each child was given a fixed time of 40 min to complete the test. Prior to the test, clear instructions were provided to both the children and their caregivers to ensure understanding.

To facilitate analysis, z scores were utilized, which were adjusted based on the children’s age to assess their intellectual aptitude. The z score was determined using the formula: z score = (IQ − average IQ)/standard deviation of IQ. Here, the average IQ represents the mean intellectual quotient for children of a specific age, and the standard deviation of IQ indicates the standard deviation for children within the same age range.

### 2.4. Statistical Analysis

A descriptive analysis was performed to investigate the demographic characteristics of the participants in this study using appropriate statistical methods. For continuous variables, such as age, mean ± standard deviations (SDs) were utilized to represent the central tendency and dispersion of the data. In our study, we ensured the normalization of these continuous variables, such as the levels of trace elements and children’s cognitive abilities, using the standard score normalization technique. We used z-score normalization, which transforms each data point in the dataset by subtracting the mean and dividing by the standard deviation, resulting in a new dataset that has a mean of 0 and standard deviation of 1. This method is particularly beneficial when the original data follows a normal distribution, as it helps in handling outliers, mitigating the impact of extreme values, and allows for easier interpretation of results.

As for the correlation analysis, Pearson’s correlation coefficients were used considering the nature of our data. (1) Type of Variables: we are dealing with continuous variables–levels of trace elements and children’s cognitive abilities. These are quantitative data that can take an infinite number of values within a given range, making them suitable for methods like Pearson correlation. (2) Distribution of Data: After normalization, our data followed a standard normal distribution, which is symmetric about the mean and characterized by its mean (0) and standard deviation (1). Pearson’s correlation is ideally used when the data are normally distributed, as it measures the linear association between variables. (3) Presence of Outliers: Prior to normalization, we examined our data for outliers and skewed distribution. The standard score normalization technique we employed helped in mitigating the impact of extreme values and made the distribution more symmetric. (4) Linearity: As Pearson’s correlation requires a linear relationship between the variables, our initial exploratory data analysis suggested a potential linear relationship between the variables of interest, which was another factor that led to the decision to use Pearson’s correlation. After the normalization process, our data followed a standard normal distribution. Pearson’s correlation is most effective and provides a more accurate result when the data are normally distributed, as it measures the linear association between variables. This method examines the degree of correlation between two continuous variables, providing insights into both the direction (positive or negative correlation) and the strength of the relationships in our data (value close to −1 or 1 indicates a strong relationship, while value close to 0 indicates a weak relationship). Hence, Pearson’s correlation was an optimal choice for our analysis in gauging the relationships between the levels of trace elements and children’s cognitive abilities.

To explore the associations between the concentrations of individual metals and children’s cognitive abilities, linear regression models were utilized. The Root Mean Square Error (RMSE) was employed as a metric to quantify the disparity between the predicted or estimated values generated by the model and the actual observed values.

To account for potential confounding variables, such as gender, maternal education level, and smoking status during pregnancy, all models were adjusted when evaluating the children’s initial cognitive abilities. IBM SPSS Statistics for Windows, Version 21.0 (IBM Corp., Armonk, NY, USA), was employed for all statistical analyses. All statistical tests were two-sided, and a significance level of *p* < 0.05 was considered.

## 3. Results

### 3.1. Study Participants’ Demographic Characteristics

In the Makkah region, we conducted interviews with 500 children free from neurological conditions. Out of these, 430 children were incorporated into this study because the parents of 70 children declined to allow blood sampling. The characteristics of the participating children are detailed in Table 2. The average age of the children included in this study was 7.57 years (SD 0.83), and 50.6% of the participants were girls. 

Table 3 describes the biochemical information of the children. Out of 430 children, 87 (20.3%) exhibited decreased iron levels, indicating iron deficiency. Zinc deficiency was found to be more prevalent, affecting 183 out of 480 children (42.5%). The copper deficiency was detected in 67 out of 480 children (14%). Among the 430 children, a deficiency in a single trace element (iron, zinc, mercury, copper) was observed in 245 individuals (56.9%). Among these children, 163 out of 245 (66.7%) had deficiencies in both iron and zinc, while a simultaneous deficiency of iron, zinc, and copper was observed in only 11 children (4.6%). The average serum iron concentration was recorded as 60.45 ± 10.64 μg/dL, and the average concentrations of zinc and copper were 76.78 ± 8.76 μg/dL and 118 ± 14.54 μg/dL, respectively.

The root-mean-square error (RMSE) for the model’s predictions on iron concentrations is 16.45 μg/dL. This signifies that the standard deviation of the residuals or prediction errors for iron is around 16.45 μg/dL, indicating a substantial deviation between the predicted and observed iron concentrations. This suggests the need for model improvement or the application of a more complex model to accurately predict iron levels. For zinc, the RMSE is calculated to be 26.78 μg/dL, indicating that our predictive model’s estimations of zinc concentrations deviate significantly from the actual observed values. This underscores the necessity for further refinement of the model to ensure accurate prediction of zinc levels. The RMSE for the predictions of copper concentrations is as high as 58 μg/dL. This large deviation is indicative of a significant discrepancy between predicted and observed copper levels. It is imperative to consider refining the model or employing a more robust predictive model to effectively predict copper concentrations. The model’s predictions for mercury concentrations result in an RMSE of 18 ng/mL. This suggests a moderate deviation between the predicted and actual observed values, suggesting that while the model provides a reasonable estimation, there still exists room for further improvement to enhance prediction accuracy. These findings underscore the necessity to refine our current predictive models or perhaps employ alternative or more complex models for effectively predicting trace element concentrations in children. 

### 3.2. Correlation of Trace Elements Intake and Children’s IQ

**Figure 1:**
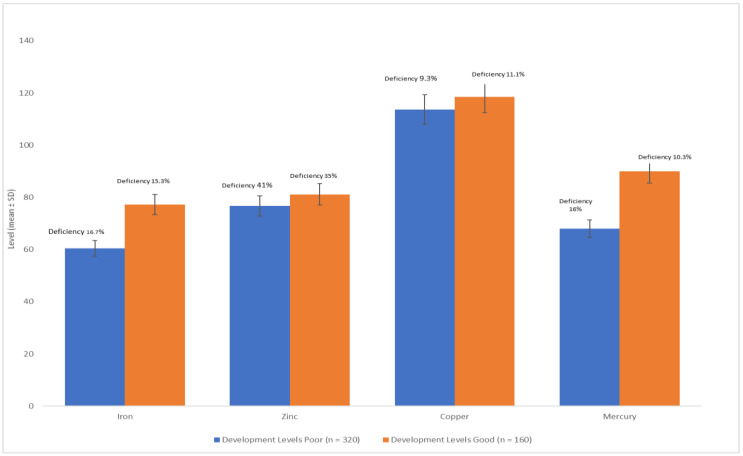
The correlation between development levels and trace element levels, as well as their deficiency levels.

The BKMR model was employed to examine the collective impact of heavy metals on continuous outcomes. A noteworthy inverse association was observed between IQ and the combined effect of heavy metals when all metal levels were above their respective median values compared to when they were at their median levels. However, no significant difference in baseline IQ was observed when all metal levels were below their median values. Intriguingly, a significant positive association was identified when the metal concentrations were below their median values. The overall relationship demonstrated statistical significance when all metals were below their 70th percentile in comparison to their median levels. Figure 1 illustrates the statistical analysis of the relationship between serum trace element concentrations and child development. Children with lower developmental levels exhibited significantly lower serum zinc levels in comparison to those with higher developmental levels (76.78 ± 10.67 vs. 81.14 ± 10.19 μg/dL), (76.79 ± 10.19 vs. 81.14 ± 10.8 μg/dL), (113.68 ± 18.67 vs. 118.45 ± 10.54 μg/dL), and (68 ± 10.54 vs. 90 ± 18.19 μg/dL) for iron, zinc, copper, and mercury, respectively. Furthermore, a higher proportion of children with lower developmental levels were found to have zinc deficiency compared to those with higher developmental levels. Although not statistically significant, children with lower developmental levels also displayed a higher prevalence of iron (16.7% vs. 15.3%), zinc (41% vs. 35%), copper (9.3% vs. 11.1%), and mercury (16% vs. 10.3%) deficiencies. The *p*-value analysis indicated an independent association between zinc deficiency and developmental levels (*p* > 0.005).

### 3.3. Correlation of Trace Elements Intake and Children’s Nutrition Habits

Table 4 displays the connection between serum levels of iron, zinc, copper, and mercury and specific eating behaviors among children. The analysis demonstrates that picky eaters had lower serum iron levels (76.59 ± 10.42 μg/dL) and higher serum copper levels (123.74 ± 13.45 μg/dL) compared to non-picky eaters. Furthermore, children with picky eating behaviors showed significantly lower zinc levels (83.37 ± 10.97 vs. 66.67 ± 9.10 μg/dL, *p* > 0.005) in comparison to those without such behaviors. Alongside reduced trace element levels, picky eating habits were also linked to a higher prevalence of trace element deficiencies. By applying RMSE values for each element in the context of picky and non-picky eating behaviors, the results for Picky Eating Behaviors (Yes) are as follows: For iron (Fe), the RMSE value of 8.8 μg/dL suggests a moderate degree of deviation between the predicted and actual iron concentrations in children exhibiting picky eating behaviors. Although this discrepancy is not exceedingly large, it indicates room for improvement in our predictive model. For zinc (Zn), with an RMSE value of 19.37 μg/dL, our predictive model displays a sizeable error margin in estimating zinc concentrations for children exhibiting picky eating behaviors. This calls for a more accurate model or adjustments to our current one. For copper (Cu), the large RMSE value of 56.74 μg/dL suggests a significant disparity between our model’s predicted copper concentrations and the observed values in picky eaters. This indicates that our current model may not be suitable for predicting copper concentrations in this subset of children. For mercury (Hg), the RMSE value of 36 ng/mL indicates a substantial deviation between the predicted and observed mercury concentrations. This large error suggests the need for a revised or entirely different predictive model. While for the Non-Picky Eating Behaviors (No), the following were noted: For iron (Fe), the RMSE value of 20.1 μg/dL signifies a sizable discrepancy between the predicted and actual iron concentrations in non-picky eaters. Despite the model’s relative accuracy, there is still scope for further improvement. For zinc (Zn), with an RMSE value of 10.67 μg/dL, our predictive model demonstrates a reasonable degree of accuracy in estimating zinc concentrations in non-picky eaters. However, there is still room for enhancing the model’s precision. For copper (Cu), an RMSE value of 21 μg/dL indicates a fair level of accuracy of our model in predicting copper concentrations in non-picky eaters. Still, there exists potential for refining our predictive model to reduce this error margin. For mercury (Hg), the relatively low RMSE value of 7 ng/mL suggests that our model fairly accurately predicts mercury concentrations in non-picky eaters. While this is encouraging, further refinement can potentially improve the model’s accuracy. In conclusion, the discrepancies highlighted by these RMSE values suggest that while our current predictive model can provide a reasonable estimate of trace element concentrations in children, it demonstrates a greater degree of accuracy for non-picky eaters. However, across both groups, there is a need for model refinement or possibly the exploration of more sophisticated models to improve predictive accuracy.

## 4. Discussion

The objective of this study was to evaluate the serum levels of trace elements (iron, zinc, copper, and mercury) in children aged 7–10 years in Saudi Arabia and investigate their correlation with selective eating habits and intellectual quotient (IQ). This study also examined the prevalence of trace element deficiencies in a sample of seemingly healthy children from the Makkah region. Zinc deficiency was found to be highly prevalent, affecting 42.5% of the children, followed by copper deficiency in 14% of the participants. Among the children, 56.9% exhibited deficiencies in at least one trace element, with 66.7% showing deficiencies in both iron and zinc. The average serum concentrations were recorded as 60.45 ± 10.64 μg/dL for iron, 76.78 ± 8.76 μg/dL for zinc, and 118 ± 14.54 μg/dL for copper. Zinc deficiency was significantly associated with selective eating habits and lower developmental levels. Non-picky eaters exhibited higher iron levels and lower copper levels in comparison to picky eaters. Furthermore, picky eating habits were correlated with a higher prevalence of trace element deficiencies.

A significant number of studies have addressed trace elements prevalence, revealing a broad range of levels in children’s blood depending on their eating preferences [6]. Most of these studies rely on the parents’ or caregivers’ assessment to classify a kid as a picky eater [42]. In our study, we utilized structured questionnaires focusing on four distinct signs of picky eating, thus providing a more objective approach. The incidence of picky eating in our study population was 56%, which is somewhat elevated compared to prior studies [6,42,43,44]. A new study proposed three main parental-reported feeding behaviors that could potentially identify persistent picky eaters at an early age [45]. These included the parent’s subjective perception of their child as a picky eater, as well as two common picky eating behaviors: a strong preference for certain foods and a reluctance to try new foods. In a comprehensive assessment of 7057 children aged between 2 and 7 years old in Hong Kong, 43% were identified by their parents as picky eaters [46]. Another research demonstrated that picky eating persists for over 2 years in 40% of children [47]. Another study focusing on Chinese preschoolers reported a higher prevalence in the 24–35-month age group (36%) compared to the 6–11-month-old group (12%) [48]. The potential nutritional deficits associated with picky eating and its impact on children’s growth status have been evaluated [49,50,51]. A longitudinal experiment involving 120 children aged between 2 and 11 years found no significant influence on growth [52]. Another cross-sectional survey examined the correlation relating to eating behaviors, including picky eating, eating adequacy, body weight, and other metrics, in 1498 children at various ages (2.5, 3.5, and 4.5 years). This study determined that picky eaters consumed significantly less energy, fats, and proteins than non-picky eaters [53]. Picky eating in preschool and early school-age children was associated with an adverse impact on growth, indicating a negative influence [54]. In a study conducted in Saudi Arabia, 315 preschool children with feeding difficulties were compared to 100 healthy controls. The findings revealed that the predominant feeding issue among the children with feeding problems was picky eating, accounting for 85.5% of the cases, although these children still displayed normal growth parameters. However, their growth metrics were significantly lower than those of healthy children [55]. In another study in China involving 937 healthy children aged 3–7 years, parents reported a 54% prevalence of picky eating. The study revealed that picky eaters had significantly lower weight-for-age z-scores compared to non-picky eaters [56].

Furthermore, our study investigated the correlation between exposure to heavy metals and IQ scores. The cumulative effects of exposure to multiple metals were assessed using Raven’s Standard Progressive Matrices. An inverse relationship was observed between IQ scores and exposure to multiple metals. Through a linear regression analysis focusing on individual metals, we observed a negative association between iron deficiency and children’s IQ. Additionally, our findings indicated that girls exhibited higher IQ levels compared to boys. This result was aligned with a study conducted by Awadh et al., 2023, which showed a connection between zinc deficiency and the occurrence of epileptic seizures, a condition frequently observed in individuals with autism. Autistic individuals are often distinguished by elevated levels of manganese and copper in their serum. Significant reductions in lead and cadmium levels in the urine of children diagnosed with autism are reported, but at the same time, chromium levels are noticeably higher. Additionally, individuals with autism spectrum disorder (ASD) have been found to have increased levels of arsenic and mercury in their blood [57]. In our previous study conducted in 2023, we discovered an independent association between children’s IQ scores and the simultaneous exposure to five trace elements present in ambient air: lead (Pb), manganese (Mn), cadmium (Cd), chromium (Cr), and arsenic (As). This study provides evidence of a correlation between combined exposure to these five heavy metals (Pb, Mn, Cd, Cr, and As) and children’s IQ scores [58], while a different study showed no difference in the timeline of reaching developmental milestones between the group receiving a placebo and the group supplemented with zinc. Furthermore, no study offered information regarding cognitive scores, Intelligence Quotients (IQs), or any potential side effects of zinc supplementation. Thus, there is no substantial proof to suggest that providing zinc supplements to infants or children leads to enhanced motor skills or cognitive development [59].

Mercury toxicity is a concerning issue in children’s health, and its potential impact on cognitive development has been investigated in various studies. Although this current study primarily focused on iron, zinc, and copper deficiencies, it is worth discussing the implications of mercury levels observed in the study population.

Within our study, the mean serum concentration of mercury was recorded as 68 ± 10.54 ng/mL, which exceeds the reference level of 100 ng/mL associated with mercury toxicity [60]. While the prevalence of mercury deficiency was not directly examined in this study, it is important to note that a significant proportion of children (16%) exhibited mercury levels above the established reference level. This finding raises concerns about potential mercury toxicity and its potential impact on children’s health and development [61,62].

Mercury, recognized as a neurotoxin, poses potential harm to the developing brain and nervous system. Children, due to their heightened exposure rates and heightened vulnerability, are particularly susceptible to the detrimental effects of mercury toxicity [63]. Studies have linked mercury exposure in children to cognitive impairments, developmental delays, and behavioral problems [64]. The observed association between mercury levels and lower developmental levels in this study further supports the potential adverse effects of mercury toxicity on children’s neurodevelopment.

Exposure to mercury can occur via various sources, including contaminated food, particularly fish and seafood, as well as environmental pollutants, such as air and water pollution [65]. Further investigations are needed to assess the specific sources and pathways of mercury exposure in this population and to develop strategies for reducing exposure and protecting children’s health.

Efforts to minimize mercury exposure and mitigate its potential adverse effects should include public health interferences, such as increasing awareness concerning the sources of mercury and providing guidance on safe fish consumption. Additionally, regular monitoring of mercury levels in children, along with comprehensive risk assessments, can help identify individuals at risk and guide targeted interventions [66].

It is crucial to acknowledge that this present study did not directly examine the precise health outcomes linked to mercury toxicity or its interplay with other trace elements. Further research is necessary to investigate the potential synergistic effects between mercury and other trace elements, as well as the enduring consequences of mercury exposure on children’s cognitive function and overall wellbeing.

To conclude, while the focus of this study was primarily on iron, zinc, and copper deficiencies, the observed levels of mercury in the study population raise concerns about potential mercury toxicity. Future studies should further investigate the sources and impact of mercury exposure in children, as well as develop strategies for minimizing exposure and protecting children’s neurodevelopment. By addressing these issues, we can work towards ensuring the optimal health and wellbeing of children in the Makkah region and beyond.

While our study provides valuable insights, there are some limitations to consider. The utilization of a cross-sectional design in this study restricts the ability to establish causal relationships between exposure and outcome variables. The reliance on parent-filled questionnaires introduces the possibility of self-report bias and limited recall accuracy. Closed-ended questions in the questionnaires may restrict the breadth of responses. Excluding children with chronic illnesses and restricting the age range may affect the generalizability of the findings. Future studies should adopt a prospective, population-based approach, encompassing children from diverse socioeconomic backgrounds nationwide.

## 5. Conclusions

In conclusion, this study offers novel insights into the prevalence of trace element deficiencies and their association with selective eating behaviors and intellectual quotient (IQ) in Saudi Arabian children. Selective eating habits, prevalent in over two-thirds of the children, were associated with lower levels of these trace elements, suggesting a potential link between dietary preferences and nutrient deficiencies. Furthermore, this study identified a significant negative correlation between collective exposure to these heavy metals and children’s IQ scores. Girls demonstrated higher IQ scores than boys, a finding in line with previous research. Future research should involve a larger, more diverse cohort of children from different socioeconomic backgrounds and aim to address these limitations via prospective, longitudinal study designs.

Ultimately, the findings underscore the importance of monitoring nutritional status in children, given the significant implications for their cognitive development and overall health. They also highlight the need for targeted interventions to manage selective eating behaviors and trace element deficiencies, as well as further research to understand these associations in greater depth.

## Figures and Tables

**Table 1:** The instrumental and data acquisition parameters employed in the ICP-Perkin Elmer 7300.

Instrumental Parameters		Data Acquisition	
RF power	1400 W	Measuring mode	Segmented scan
Argon gas	flow 13–16 L/min	Point per peak	5
Nebulizer	1.0 L/min	Scans/replicates	3
Plasma	18.0 L/min	Replicate/sample	3
Sample	rate 190 s	Integration time	398.6 s

**Table 2:** Summary data for study population.

Studied Participants	Total (*n* = 430)	*p*-Value
	Mean ± SD	
Age (years)	7.38 ± 0.89	<0.001
Gender Boys Girls	212 (49.4%) 218 (50.6%)	>0.05
Body Mass Index (kg/m^2^)	15.94 ± 2.40	>0.05
Mother Education Level		>0.05
Primary Level	101 (23.7%)	
High School	272 (63.3%)	
University Level	57 (13%)	
Smoking During Pregnancy No Yes	414 (96.5%) 16 (3.5%)	<0.001
Selective Eating	334 (77.6%)	>0.05

Abbreviation: SD, standard deviation. *p*-values for age and Body Mass Index were computed using a one-sample *t*-test, assuming a normal distribution. For categorical variables such as gender, mother’s education level, smoking during pregnancy, and selective eating, *p*-values were computed using a chi-squared test to compare observed frequencies with expected frequencies. We ensured that the assumptions of the test are met by data.

**Table 3:** Biochemical data and predictive error for children with normal and deficient micronutrient levels.

Parameter	Values	RMSE	Deficiency Level [41]
	Mean ± SD		
Iron (Fe)	60.45 ± 10.64	16.45	<50 μg/dL
Zinc (Zn)	76.78 ± 8.76	26.78	<70 μg/dL
Copper (Cu)	118 ± 14.54	38.34	<90 μg/dL
Mercury (Hg)	68 ± 10.54	18.23	>100 ng/mL

Abbreviation: SD, standard deviation; RMSE, Root Mean Squared Error.

**Table 4:** Association of trace elements concentration and children’s nutritional habits.

Parameter	Picky Eating Behaviors				*p*-Value
	Yes (*n* = 334, 77.6%) Mean ± SD	RMSE	No (*n* = 96, 22.3%) Mean ± SD	RMSE	
Iron (Fe)	66.8 ± 10.10	8.8	80.1 ± 23.45	20.1	>0.005
Zinc (Zn)	83.37 ± 10.97	19.37	66.67 ± 9.10	10.67	>0.005
Copper (Cu)	123.74 ± 13.45	56.74	87 ± 4.10	21	>0.005
Mercury (Hg)	76 ± 12.64	36	90 ± 23.54	7	>0.005

Abbreviation: SD, standard deviation; RMSE, Root Mean Squared Error. *p*-values were calculated using an independent two-sample *t*-test, comparing mean trace element concentrations between groups of children with and without picky eating behaviors.

## Data Availability

The data presented in this study are available on request from the corresponding author.
